# Approaching autozygosity in a small pedigree of Gochu Asturcelta pigs

**DOI:** 10.1186/s12711-023-00846-7

**Published:** 2023-10-25

**Authors:** Katherine D. Arias, Juan Pablo Gutiérrez, Iván Fernández, Isabel Álvarez, Félix Goyache

**Affiliations:** 1 Área de Genética y Reproducción Animal, SERIDA-Deva, Camino de Rioseco 1225, 33394 Gijón, Spain; 2 Departamento de Producción Animal, Universidad Complutense de Madrid, Avda. Puerta de Hierro S/N, 28040 Madrid, Spain

## Abstract

**Background:**

In spite of the availability of single nucleotide polymorphism (SNP) array data, differentiation between observed homozygosity and that caused by mating between relatives (autozygosity) introduces major difficulties. Homozygosity estimators show large variation due to different causes, namely, Mendelian sampling, population structure, and differences among chromosomes. Therefore, the ascertainment of how inbreeding is reflected in the genome is still an issue. The aim of this research was to study the usefulness of genomic information for the assessment of genetic diversity in the highly endangered Gochu Asturcelta pig breed. Pedigree depth varied from 0 (founders) to 4 equivalent discrete generations (*t*). Four homozygosity parameters (runs of homozygosity, *F*_*ROH*_; heterozygosity-rich regions, *F*_*HRR*_; Li and Horvitz’s, *F*_*LH*_; and Yang and colleague’s *F*_*YAN*_) were computed for each individual, adjusted for the variability in the base population (BP; six individuals) and further jackknifed over autosomes. Individual increases in homozygosity (depending on *t*) and increases in pairwise homozygosity (i.e., increase in the parents’ mean) were computed for each individual in the pedigree, and effective population size (*N*_*e*_) was computed for five subpopulations (cohorts). Genealogical parameters (individual inbreeding, individual increase in inbreeding, and *N*_*e*_) were used for comparisons.

**Results:**

The mean *F* was 0.120 ± 0.074 and the mean BP-adjusted homozygosity ranged from 0.099 ± 0.081 (*F*_*LH*_) to 0.152 ± 0.075 (*F*_*YAN*_). After jackknifing, the mean values were slightly lower. The increase in pairwise homozygosity tended to be twofold higher than the corresponding individual increase in homozygosity values. When compared with genealogical estimates, estimates of *N*_*e*_ obtained using *F*_*YAN*_ tended to have low root-mean-squared errors. However, *N*_*e*_ estimates based on increases in pairwise homozygosity using both *F*_*ROH*_ and *F*_*HRR*_ estimates of genomic inbreeding had lower root-mean-squared errors.

**Conclusions:**

Parameters characterizing homozygosity may not accurately depict losses of variability in small populations in which breeding policy prohibits matings between close relatives. After BP adjustment, the performance of *F*_*ROH*_ and *F*_*HRR*_ was highly consistent. Assuming that an increase in homozygosity depends only on pedigree depth can lead to underestimating it in populations with shallow pedigrees. An increase in pairwise homozygosity computed from either *F*_*ROH*_ or *F*_*HRR*_ is a promising approach for characterizing autozygosity.

**Supplementary Information:**

The online version contains supplementary material available at 10.1186/s12711-023-00846-7.

## Background

In small populations in which preservation of genetic variability is the goal, possible losses of genetic diversity have been traditionally assessed using genealogical information by computing coefficients of inbreeding (*F*) [1], defined as the probability that two alleles sampled at random at a locus are identical-by-descendent (IBD), or coefficients of coancestry (*f*) [2], defined as the probability that two alleles sampled at random in two different individuals are IBD. However, how *F* and *f* coefficients are reflected in the genome remains unclear. Although genealogical analyses assume that each individual in the base population has unique alleles at each locus, genomic data are subject to finite sampling and, thus, homozygosity at a locus can occur by chance. In other words, two alleles at a locus can be identical-by-state (IBS) rather than IBD. Therefore, estimating homozygosity due to IBD in an individual (autozygosity sensu, Cotterman [3]) has major difficulties because estimates widely vary from expectations as a consequence of Mendelian sampling [4] and the variance in IBD sharing per chromosome [5]. In this scenario, genomic estimates of homozygosity tend to measure the ‘realized inbreeding’ of an individual rather than the effect of autozygosity [6].

Ascertainment of the relationships between genealogical and genomic estimates of *F* has usually been approached using either simulated [7–13] or real [14–17] data with deep pedigrees. However, inconsistencies between these two sources of information may be greater in the case of small populations with shallow pedigrees and high proportions of full and half-siblings. Such scenarios could lead to weak correlations between pedigree-based and genomic estimates of inbreeding [8]. Moreover, mating policies can also affect the informativeness of genomic estimators. When matings between close relatives are avoided, allele frequencies tend to remain balanced across years and generations [18, 19]. In such scenarios, parameters that characterize homozygosity may not show clear trends [15].

There is consensus that the use of genomic estimators of inbreeding (homozygosity) may be beneficial to conservation programmes by allowing them to maintain the largest possible amount of genetic diversity [20, 21]. However, their efficiency could be affected by the genotyping platform[10, 19, 22], as well as by the characteristics of the population [13, 23–25]. Furthermore, genomic coefficients of inbreeding are highly dependent on the assumptions underlying their definition and do not always provide useful estimates of inbreeding due to inconsistent results in terms of gain and loss of genetic variability [15].

The rate of inbreeding computed using genealogical information is a standard criterion considered in conservation programmes, as it translates to the effective population size (*N*_*e*_) of a population [22, 26]. It can be efficiently estimated using the individual increase in inbreeding coefficients ($\Delta {F}_{i}$) [27, 28]. The parameter $\Delta {F}_{i}$ is not affected by changes in mating policy, allows a flexible definition of the reference population in a pedigree, and gives useful estimates of *N*_*e*_ even with shallow pedigrees [28, 29]. However, similar approaches have not been attempted using genomic data.

The Gochu Asturcelta breed is an extremely endangered Celtic–Iberian pig population [30, 31] for which a conservation programme was started with six founders, only four of which provided viable offspring [32]. The start of the recovery programme has been well documented [32, 33]: its first stages were affected by incorrect management practices, including full-sib matings [34], causing a sudden increase in inbreeding. Immediately thereafter, a strict mating policy prohibiting matings between close relatives and prolonging the reproductive career of the founders and their direct descendants as much as possible was implemented to keep founder contributions balanced across generations and to preserve their genetic background in the present population [32]. The mating policies generated a complex pedigree useful for testing different hypotheses on the relationship between pedigree and genomic information and the preservation of genetic diversity [32, 33, 35].

The main objective of this research was to obtain empirical evidence on the usefulness of genomic information for the assessment of genetic diversity in small populations with shallow pedigrees using a sample of Gochu Asturcelta pigs. The performance of four estimators of homozygosity and the influence of genomic variability in the base population will be assessed, as well as the partitioning of genomic diversity into autosomes. An increase in homozygosity will be estimated at the individual level following two approaches: (a) computation of individual increases in homozygosity as an extension of the genealogical approach proposed by Gutiérrez et al. [27] and (b) computation of the increase in homozygosity of an individual over the mean homozygosity of its parents. Based on these estimates of increase in homozygosity, genomic estimates of *N*_*e*_ will be computed and further compared with genealogical estimators ($\Delta {F}_{i}$ and realized *N*_*e*_). Insights for programmes aimed at the preservation of genetic variability in small animal populations will be discussed.

## Methods

SERIDA is associated with the Ethical Committee in Research of the University of Oviedo (Spain), which ensures that all research with biological agents follows Good Laboratory Practices and European and Spanish regulations on biosecurity (Regulation of February 13th, 2014; BOPA no.47, February 26th, 2014). Tissue and hair root samples used in this project were collected by veterinary practitioners working for the Gochu Asturcelta Breeders’ Association (ACGA), with the permission and in the presence of the owners of the animals. For this reason, permission from the Ethical Committee in Research of the University of Oviedo was not required. In all instances, ACGA veterinarians followed standard procedures and relevant national guidelines to ensure appropriate animal care.

### Samples and genotyping

The analysed pedigree included 534 genotyped individuals, forming 526 parents–offspring trios, corresponding to 76 families (descendants of the same boar–sow pair) and 95 litters registered between 2000 and 2010. Data include the genotypes of 24 boars, 42 sows, and their offspring. Family size (offspring per family) varied from 1 to 34, with 48 families having 5 offspring or more. Based on pedigree, up to 105 genotyped individuals were noninbred (*F* = 0). Two genotyped founders and four full-sib individuals (born from 2003 to 2005 and direct descendants from two non-genotyped founders with offspring in the dataset [32]) were considered as the base population (BP); their genotypes were used to calculate genomic estimators of inbreeding relative to the BP.

Individuals were genotyped with Axiom-PorcineHDv1 of Affymetrix (658,692 single nucleotide polymorphisms, SNPs). The software Axiom Analysis Suite v4.0.3 (Thermo Fisher Scientific, Waltham, MA, USA) was used to create standard.ped and.map files. The genotyped SNPs were mapped using the *Sscrofa* genome build 11.1 [36]. Only SNPs on autosomal chromosomes with known positions were considered. To avoid the presence of null and false alleles, genotypes were only filtered on the basis of Mendelian errors [37]. In total, 503,043 SNPs (151,226 of them monomorphic) with a minimum call rate of 0.97 were retained for further analysis.

### Pedigree analyses

The pedigree data were analysed using the program ENDOG v4.8 [38]. The pedigree was described by computing the number of fully traced generations (*G*) and the equivalent discrete generations ($t$) for each individual. *G* is defined as the number of generations separating the offspring from the farthest generation where the two ancestors of the individual are known, while $t$ is the sum of (1/2)^*n*^, where $n$ is the number of generations separating the individual from each known ancestor [39].

Inbreeding coefficients (${F}_{i}$) were computed for each individual $i$ in the pedigree following Meuwissen and Luo [40]. The increase in inbreeding ($\Delta {F}_{i}$) was computed as proposed by Gutiérrez et al. [28] as $\Delta {F}_{i}=1-\sqrt[{t}_{i}-1]{\left(1-{F}_{i}\right)}$. The realized effective population size was computed for each cohort by averaging the individual increase in inbreeding coefficients of the $n$ individuals included in the cohort as ${Ne}_{{F}_{i}}=1/2\overline{\Delta {F }_{i}}$ [27, 29]. As a complementary approach, *N*_*e*_ was also computed from the increase in pairwise coancestry [41] as ${Ne}_{{C}_{ij}}=1/(2\overline{\Delta C })$, with $\overline{\Delta C }$ being the mean value of the increase in pairwise coancestry $\Delta {C}_{ij}=1-\sqrt[{(t}_{i}+{t}_{j})/2]{(1-{C}_{ij})}$, where ${C}_{ij}$ is the coancestry coefficient between individuals $i$ and $j$ and ${t}_{i}$ and ${t}_{j}$ are their respective equivalent discrete generations.

### Reference subpopulations

For descriptive purposes, most analyses were presented for both the whole genotyped population and the subpopulation of individuals with two or more equivalent discrete generations ($t$) in their pedigree ($t$-subset; 480 individuals). Furthermore, different cohorts were defined according to (a) the year of birth (C2007, 112 offspring; C2008, 208 offspring; and C2009, 125 offspring, including 20 individuals born in 2010) and (b) the number of complete generations in their pedigree (two complete generations—CG2, 325 offspring; and three complete generations—CG3, 149 individuals).

### Estimates of homozygosity

Four parameters that characterize homozygosity (${F}_{ROH}$, ${F}_{HRR}$, ${F}_{LH}$, and ${F}_{YAN}$) were computed:

- ${F}_{ROH}$ is the proportion of the genome covered by stretches of homozygous sites, usually referred to as runs of homozygosity (ROH) [42]. ${F}_{ROH}$ for individual $i$ was computed as ${F}_{{ROH}_{i}}=\frac{\sum {L}_{{ROH}_{i}}}{{L}_{AUTO}}$, where $\sum {L}_{{ROH}_{i}}$ is the length of the genome covered by ROH in individual $i$ and ${L}_{AUTO}$ is the length of the autosomal genome [42]. Identification of ROH for each individual was carried out using the consecutive runs approach implemented in the package detectRuns in R [43], using the following parameters: a maximum number of five heterozygous SNPs and a maximum number of five missing SNPs in a run; a minimum number of 20 SNPs in a run; a minimum length of 1 kb; and a maximum distance of 1 Mb allowed between consecutive SNPs. These parameters are expected to allow almost complete detection of ROH [44] and to avoid bias caused by recombination events [45]. For descriptive purposes only, the ROH segments identified in each individual were summarized into ROH regions using the *intersectBed* function of the BedTools version 2.28.0 software [46], and the results were plotted as an idiogram using the package RIdeogram of R [47].
- ${F}_{HRR}$ is the proportion of the genome covered by heterozygosity-rich regions (HRR; formerly known as runs of heterozygosity) [48]. ${F}_{HRR}$ for individual $i$ was computed as ${F}_{{HRR}_{i}}=1-\frac{\sum {L}_{{HRR}_{i}}}{{L}_{AUTO}}$, where $\sum {L}_{{HRR}_{i}}$ is the length of the genome covered by HRR in individual $i$. Identification of HRR for each individual was performed using the consecutive runs approach implemented in the package detectRuns in R [43]. Given that HRR segments are expected to be less frequent and shorter (< 1 Mb) than ROH and that the main parameter leading to their identification is the number of homozygous SNPs allowed in a segment [17, 44, 49], the following parameters were used to detect HRR: a maximum number of five homozygous SNPs in a run; a maximum number of five missing SNPs in a run; a minimum number of 10 SNPs in a run; a minimum length of 1 kb; and a maximum distance of 1 Mb allowed between consecutive SNPs. For descriptive purposes, the HRR segments identified in each individual were summarized into HRR regions using the *intersectBed* function of the BedTools version 2.28.0 software [46], and the results were plotted as an idiogram using the package RIdeogram of R [47].
- ${F}_{LH}$ is the Li and Horvitz [50] estimator of the deviation of the observed frequency of homozygotes from that expected in the BP under Hardy–Weinberg proportions. The diagonals of the Li and Horvitz [50] relationship matrix were computed using custom R code as ${F}_{LH}=\frac{{SF}_{NEJ}-\sum_{k=1}^{S}\left[1-{2p}_{k\left(0\right)}(1-{p}_{k\left(0\right)})\right]}{S-\sum_{k=1}^{S}\left[1-{2p}_{k\left(0\right)}(1-{p}_{k\left(0\right)})\right]}$, where $S$ is the number of loci, ${F}_{NEJ}$ is the proportion of loci homozygous in individual $i$ [51] and ${p}_{k(0)}$ is the frequency of the reference allele of SNP $k$ in the BP. ${F}_{LH}$ is equivalent to ${F}_{IS}$ [52, 53] after correction for the homozygosity in the BP and, therefore, gives information in terms of IBD.
- ${F}_{YAN}$ is equal to the diagonals of the genomic relationship matrix constructed using the Yang et al. [54] approach based on the correlation between uniting gametes. Using custom R code, ${F}_{YAN}$ was computed as ${F}_{YAN}=\frac{1}{S}{\sum }_{k=1}^{S}\frac{{x}_{k}^{2}-(1+2{p}_{k(0)}){x}_{{k}_{i}}+2{p}_{k(0)}^{2}}{2{p}_{k(0)}(1-{p}_{k(0)})}$, where ${x}_{k}$ is the genotype of the individual for SNP $k$. Yang et al.’s [54] estimator gives more weight to homozygotes for the minor allele than to homozygotes for the major allele.

The four parameters computed are expected to characterize differences: (a) in the length of homozygous genomic stretches (${F}_{ROH}$); (b) in the length of non-random heterozygous genomic segments (${F}_{HRR}$); (c) in expected homozygosity (${F}_{LH}$); and (d) on the diagonal of the genomic relationship matrix (${F}_{YAN}$). Unlike ${F}_{ROH}$ and ${F}_{HRR}$, both ${F}_{LH}$ and ${F}_{YAN}$ account for allele frequencies in a previously defined BP.

For descriptive purposes, possible trends in homozygosity estimates by pedigree depth ($t$) were assessed by computing regression coefficients using the package STAT of R [55].

### Adjustment of estimates of homozygosity

To improve robustness in the estimates of ${F}_{ROH}$, ${F}_{HRR}$, ${F}_{LH}$ and ${F}_{YAN}$ for each individual in the dataset, two consecutive adjustment strategies were applied:

- Following Powell et al. [56], individual estimates of homozygosity were adjusted for the mean homozygosity in the BP as ${F}_{ia}=\frac{{F}_{i}-{F}_{BP}}{1-{F}_{BP}}$, where ${F}_{ia}$ is the individual homozygosity estimate adjusted for the BP, ${F}_{i}$ is the homozygosity estimate (${F}_{ROH}$, ${F}_{HRR}$, ${F}_{LH}$ or ${F}_{YAN}$) for individual $i$ and ${F}_{BP}$ is the mean of the BP for each corresponding homozygosity estimate. Note that this BP adjustment is independent of the use of the allele frequencies in the BP for the computation of ${F}_{LH}$ and ${F}_{YAN}$.
- Considering the partitioning of genomic diversity into chromosomes, BP-adjusted estimates were also computed for each autosome and further averaged at the individual level by jackknifing over autosomes [57].

Hereafter, references to unadjusted estimates will be referred to as ‘raw’ estimates, estimates adjusted for the mean homozygosity in the BP will be referred to as ‘BP-adjusted’ estimates, and those computed with additional jackknifing over autosomes as ‘jackknifed’ estimates.

### Increases in genomic inbreeding and ***N***_***e***_

For the four parameters used, the increase in homozygosity was computed at the individual level in two ways:

- Individual increase in homozygosity, computed as $\Delta t{F}_{i}=1-\sqrt[t]{1-{F}_{i}}$, where ${F}_{i}$ is either the BP-adjusted or the jackknifed estimate of homozygosity and $t$ is the equivalent discrete generations in the pedigree of the individual.
- Increase in pairwise homozygosity, computed as $\Delta p{F}_{ijk}=\frac{{F}_{i}-\left[0.5\left({F}_{j}+{F}_{k}\right)\right]}{1-\left[0.5\left({F}_{j}+{F}_{k}\right)\right]}$, where ${F}_{i}$, ${F}_{j}$ and ${F}_{k}$ are either the BP-adjusted or the jackknifed estimates of homozygosity of offspring $i$, parent $j$, and parent $k$, respectively.

Note that both these approaches are applied for each individual in the dataset as the deviations from either an “ideal” population with $t$ = 0 (individual increase in homozygosity) or the mean genomic inbreeding of the parents of the individual, assumed to be a “reference population” from which each offspring is differentiated (increase in pairwise homozygosity).

Effective population sizes were subsequently computed by averaging $\Delta t{F}_{i}$ and $\Delta p{F}_{ijk}$ among all individuals in a cohort and computed as ${\overline{N}}_{{e}_{{F}_{i}}}=\frac{1}{2\overline{\Delta t{F}_{i}}}$ and ${\overline{N}}_{{e}_{{F}_{ijk}}}=\frac{1}{2\overline{\Delta p{F}_{ijk}}}$, respectively. Uncertainty of the ${\overline{N}}_{{e}_{{F}_{i}}}$ and ${\overline{N}}_{{e}_{{F}_{ijk}}}$ estimates obtained for the two yearly and genealogical cohorts defined were measured via root-mean-squared error (RMSE) as $\mathrm{RMSE}=\sqrt{\frac{\sum {\left({N}_{e}-{\overline{N}}_{{e}_{F}}\right)}^{2}}{n}}$, where *N*_*e*_ is either the estimate of ${N}_{e}{F}_{i}$ or of ${N}_{e}{C}_{ij}$ obtained from genealogical information, ${\overline{N}}_{{e}_{F}}$ is the corresponding estimate of effective population size computed from either $\Delta t{F}_{i}$ or $\Delta p{F}_{ijk}$ for ${F}_{ROH}$, ${F}_{HRR}$, ${F}_{LH}$ or ${F}_{YAN}$, and $n$ is the sample size (5, in this case).

## Results

A full description of the pedigree of the Gochu Asturcelta pig used is given in association with the estimates of genomic parameters computed for each individual as Additional files (see Additional file 1: Tables S1, S2, S3, S4). Table 1 gives mean values for the genealogical parameters describing the pedigree. The mean pedigree-based inbreeding level of the population was 0.120 ± 0.074, with a mean pedigree depth of 2.8 ± 0.7 equivalent discrete generations ($t$). These values were only slightly higher (mean ${F}_{i}$ = 0.134 ± 0.066; $t$ = 2.9 ± 0.5) for the $t$-subset, which included 90% of the individuals in the dataset.

**﻿Table 1:**
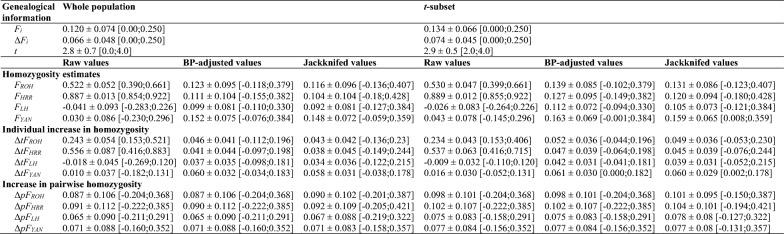
Mean (± standard deviation) of genealogical parameters (inbreeding, *F*_*i*_, individual increase in inbreeding, Δ*F*_*i*_, and equivalent discrete generations, *t*) and estimates of homozygosity (${F}_{ROH}$, ${F}_{HRR}$, ${F}_{LH}$_*,*_ and ${F}_{YAN}$) and their increase computed from genomic information

Minimum and maximum values are given in brackets. Genomic parameters are presented as raw values as well as values adjusted for the mean homozygosity in the base population (BP) and values obtained via jackknifing over autosomes. Data are given for the whole population as well as for the individuals with two or more equivalent discrete generations in their pedigree (t-subset). ΔtF values correspond to the individual increase in inbreeding computed for each of the four homozygosity parameters tested, while ΔpF values indicate the corresponding increase in pairwise homozygosity values

### Raw homozygosity estimates

**Fig. 1:**
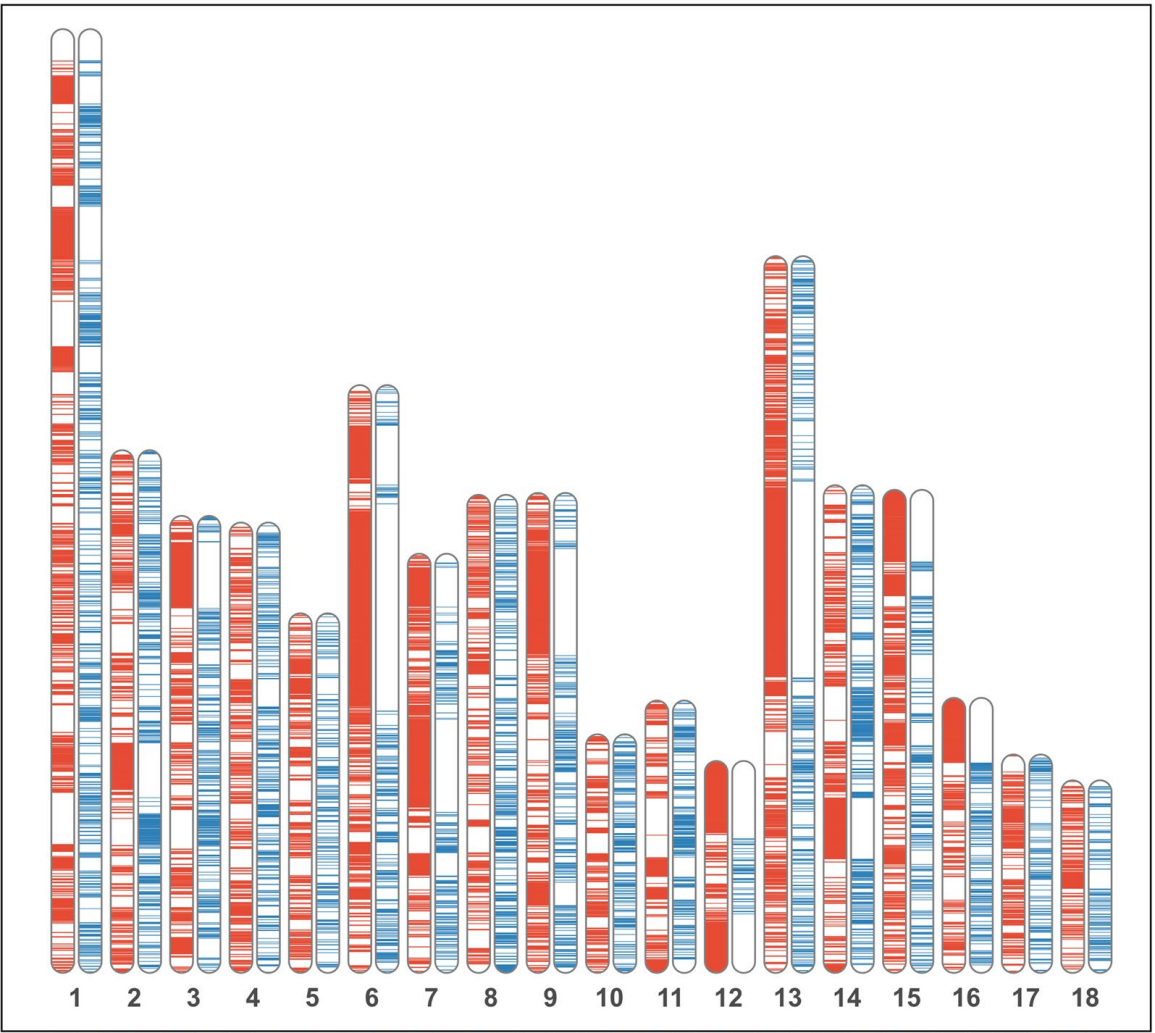
Ideogram illustrating, per autosome, the distribution of the 3017 and 4646 genomic areas in which ROH (in red) and HRR (in blue) segments were identified across the 534 genotyped Gochu Asturcelta individuals

In total, 1,411,686 and 2,176,412 ROH and HRR segments, respectively, were identified across the 534 genotyped individuals. These segments were summarized into 3017 ROH areas covering 1.04 Gb and 4646 HRR areas covering 290.7 Mb over the 18 autosomes (Fig. 1).

**Fig. 2:**
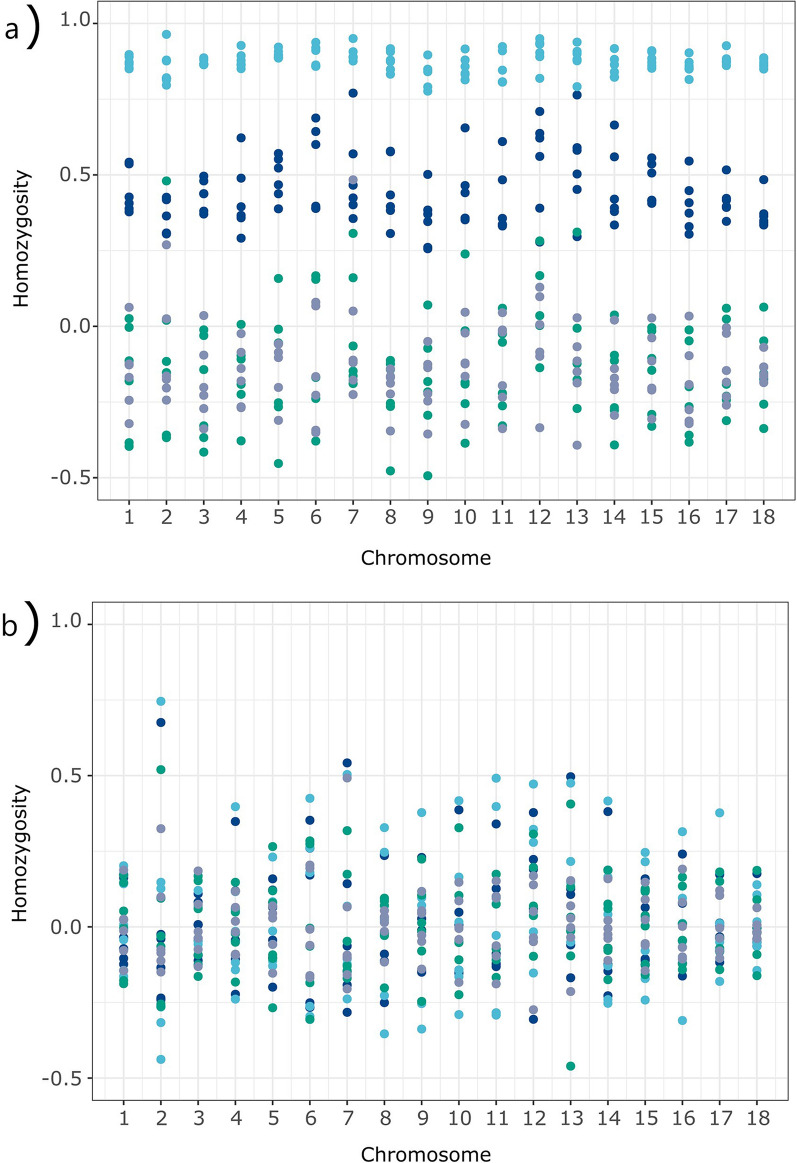
Dispersion, over the 18 autosomes, of the four estimates of homozygosity tested (${F}_{ROH}$, in dark blue; ${F}_{HRR}$, in light blue; ${F}_{LH}$, in grey; and ${F}_{YAN}$ in green) in the six individuals of the Gochu Asturcelta pedigree used as the base population. Plot (**a**) illustrates the raw mean values, whereas plot (**b**) illustrates the mean values after adjustment for the mean for each autosome in the base population

Figure 2a illustrates the dispersion of the raw estimates of homozygosity by autosome in the individuals belonging to the BP. The raw estimates showed wide dispersion and evident differences in scale among homozygosity parameters. Raw estimates of the four parameters used (${F}_{ROH}$, ${F}_{HRR}$, ${F}_{LH}$ and ${F}_{YAN}$) computed at the individual level are given in Additional file 1: Table S1 and summarised in Table 1. As expected, before BP adjustment of the estimates for each individual, ${F}_{ROH}$ and ${F}_{HRR}$ always took positive and high mean values (0.522 and above, [see Additional file 1: Table S3]). However, both ${F}_{LH}$ and ${F}_{YAN}$ took significantly lower (${F}_{YAN}$ = 0.030 ± 0.086) and even negative (${F}_{LH}$ = − 0.041 ± 0.093) mean values. Raw estimates for both ${F}_{LH}$ and ${F}_{YAN}$ took negative values in a substantial number of individuals (355 individuals, 66%, and 203 individuals, 38%, respectively), including those belonging to the BP.

### BP-adjusted homozygosity estimates

Figure 2b illustrates the effect of adjustment for the mean variability in the BP on the estimates of homozygosity, by autosome, in the BP individuals. BP adjustment gave less dispersed estimates across chromosomes. However, some individuals still had estimates of homozygosity departing from expectations on some chromosomes (namely, *Sus scrofa* (SSC) chromosome SSC2, SSC12 and SSC13).

Genomic parameters computed after adjustment for the mean homozygosity in the BP are given for each individual in the pedigree in Additional file 1: Table S2. Mean values for genomic parameters after adjustment for variability in BP are in Table 1 for both the whole population and the *t*-subset. After adjustment, both ${F}_{ROH}$ and ${F}_{HRR}$ took negative values for some individuals belonging to the first generations of the pedigree (57 individuals, 11%, and 96 individuals, 18%, respectively), whereas the frequency of individuals with negative values for ${F}_{LH}$ and ${F}_{YAN}$ was lower (73 individuals, 14%, and 11 individuals, 2%, respectively). However, the means for ${F}_{ROH}$ (0.123 ± 0.095), ${F}_{HRR}$ (0.111 ± 0.104), ${F}_{LH}$ (0.099 ± 0.081), and ${F}_{YAN}$ (0.152 ± 0.075) were always positive. The corresponding means for the $t$-subset were slightly higher, ranging from 0.112 ± 0.072 (${F}_{ROH}$) to 0.163 ± 0.069 (${F}_{YAN}$).

**Fig. 3:**
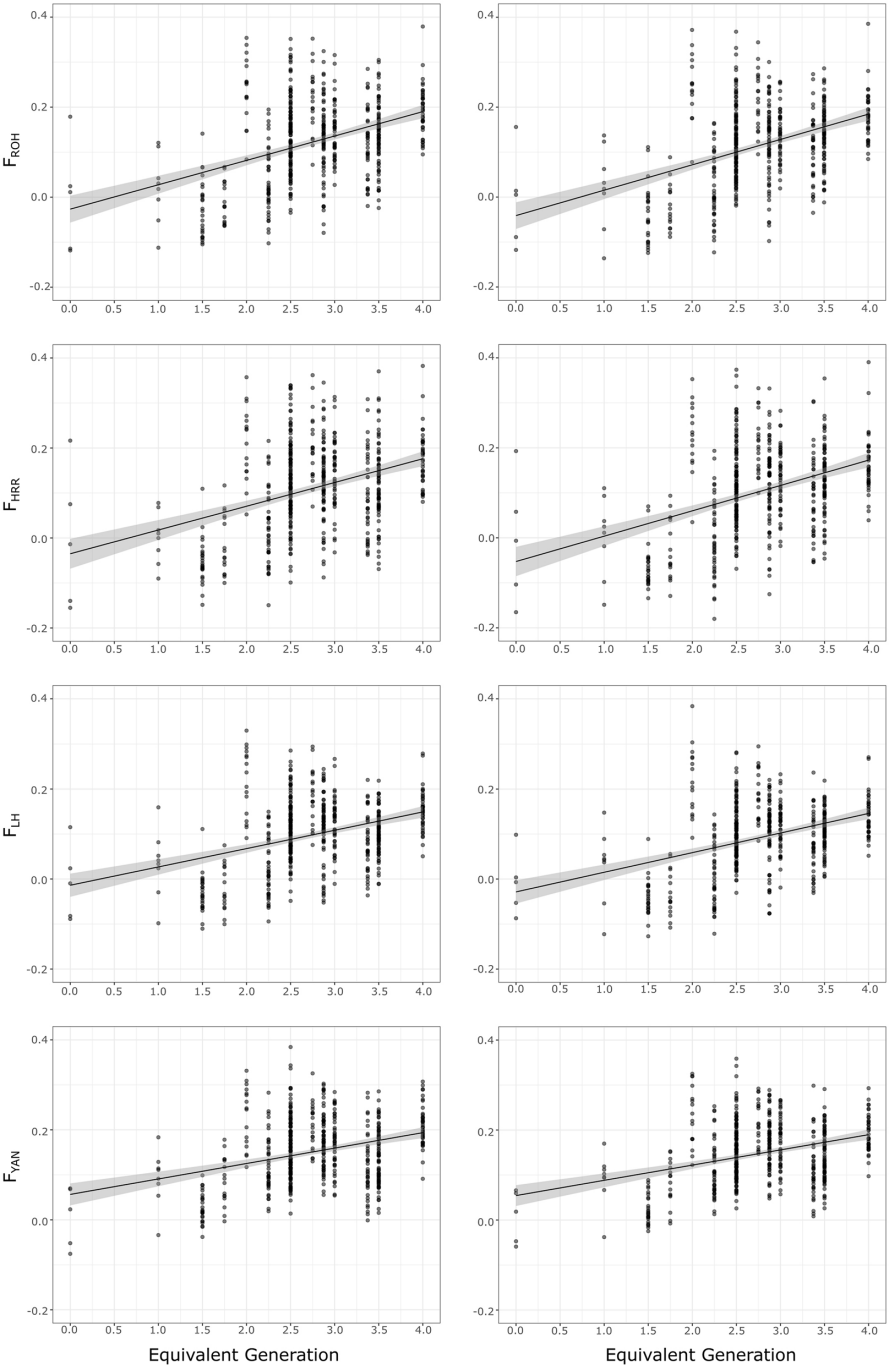
Individual variation in the four different homozygosity estimates used (${F}_{ROH}$, ${F}_{HRR}$, ${F}_{LH}$_*,*_ and ${F}_{YAN}$). Values computed for each individual in the dataset (in circles) are plotted by pedigree depth (equivalent discrete generations) of the animals. Regression of the individual homozygosity values over pedigree depth ($t$) is also displayed. The corresponding regression coefficients (*b*) and their statistical significance (*p*) are also given. The left column illustrates the variation in the base population-adjusted values, whereas the right column illustrates the variation in the jackknifed values

Figure 3 (left column) illustrates the variation in the BP-adjusted individual homozygosity values per $t$. The four homozygosity parameters showed a large amount of variation. When families with five or more offspring were considered, the within-family coefficient of variation ranged from 11 to 519% for ${F}_{ROH}$, from 17 to 334% for ${F}_{HRR}$, from 10 to 1,366% for ${F}_{LH}$, and from 55 to 1,688% for ${F}_{YAN}$. Although the homozygosity values did not show clear trends, regression of individual homozygosity estimates on *t* indicated that ${F}_{ROH}$, ${F}_{HRR}$, ${F}_{LH}$ and ${F}_{YAN}$ tended to increase significantly (*p* < 0.001) with pedigree depth. However, this scenario was not confirmed in the $t$-subset [see Additional file 1: Table S3]: regression of both ${F}_{LH}$ and ${F}_{YAN}$ estimates on $t$ became statistically non-significant (*p* > 0.10), while those corresponding to ${F}_{ROH}$ and ${F}_{HRR}$ continued to be significant (*p* < 0.05).

### Jackknifed estimates of homozygosity

Genomic parameters computed via jackknifing over autosomes are given for each individual in the pedigree in Additional file 1: Table S4. The corresponding mean values for the whole population and the $t$-subset are also in Table 1. After jackknifing, mean values were slightly lower than those obtained adjusting for BP only. A higher mean was obtained for ${F}_{YAN}$ (0.148 ± 0.072), and a lower mean was obtained for ${F}_{LH}$ (0.092 ± 0.081), whereas the means for ${F}_{ROH}$ (0.116 ± 0.096) and ${F}_{HRR}$ (0.104 ± 0.104) had intermediate values. The ranges (maximum and minimum values) of the jackknifed values computed for ${F}_{ROH}$, ${F}_{HRR}$, and ${F}_{LH}$ were higher than those computed after BP adjustment only. However, the range for ${F}_{YAN}$ was narrower than that observed after correcting for variability in the BP only.

Figure 3 also illustrates variation in the individual homozygosity values per $t$ after jackknifing over autosomes. Compared with the BP-adjusted estimates, no clear differences in variation could be assessed. However, both regression coefficients and goodness-of-fit (R^2^) of the regressions of homozygosity estimates on $t$ were slightly higher than those obtained for the BP-adjusted values (Fig. 3; Additional file 1: Table S3). The within-family coefficients of variation (for families with five or more offspring) ranged from 11 to 726% for ${F}_{ROH}$, from 17 to 723% for ${F}_{HRR}$, and from 7 to 1865% for ${F}_{LH}$. However, the range of within-family coefficients of variation for ${F}_{YAN}$ was significantly narrower (ranging from 7 to 178%).

### Increases in inbreeding and homozygosity

**Fig. 4:**
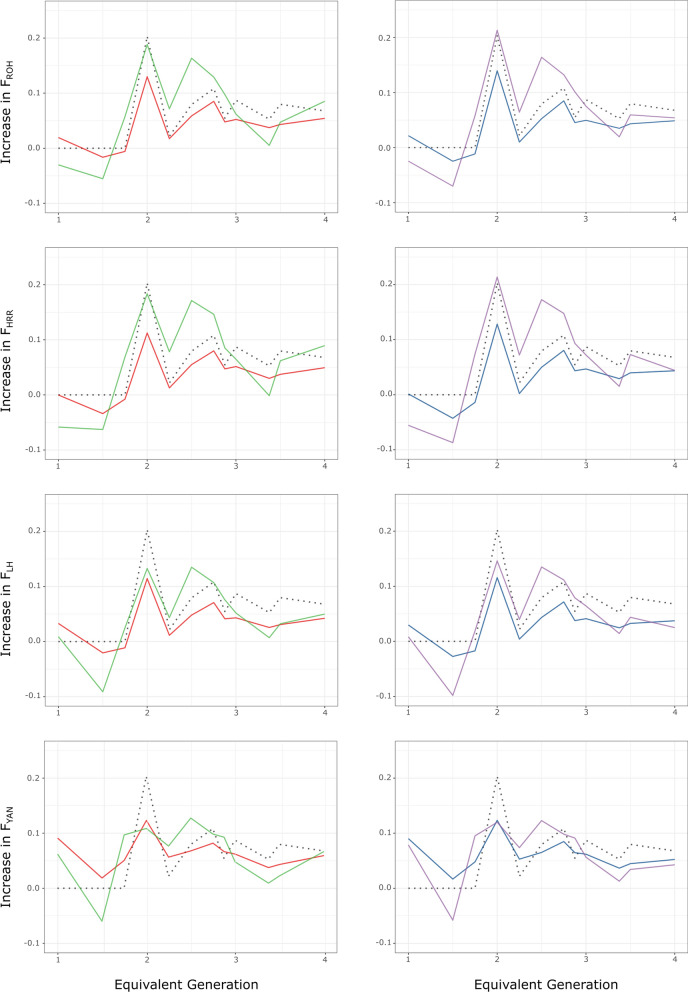
Comparison between the variation of different estimates of individual increase in homozygosity and the genealogical individual increase in inbreeding (dotted line) by pedigree depth (equivalent discrete generations; $t$). In the left column, the individual increase in homozygosity (red line) and the increase in pairwise homozygosity (green line) computed from the base population-adjusted values of individual homozygosity are given for each of the four homozygosity parameters (${F}_{ROH}$, ${F}_{HRR}$, ${F}_{LH}$_*,*_ and ${F}_{YAN}$) tested. In the right column, the individual increase in homozygosity (blue line) and the increase in pairwise homozygosity (purple line) computed from the values of individual homozygosity obtained via jackknifing over autosomes are given for the same parameters

Table 1 also gives mean values for the genealogical individual increase in inbreeding ($\Delta {F}_{i}$), as well as for the individual increase in homozygosity ($\Delta t{F}_{i}$) and the increase in pairwise homozygosity ($\Delta p{F}_{ijk}$) values computed from genomic data. Although they were computed via jackknifing over autosomes or adjusting for the variability in the BP only, mean $\Delta p{F}_{ijk}$ values were roughly twofold higher than the corresponding $\Delta t{F}_{i}$ values, except for ${F}_{YAN}$_*,*_ for which the mean values were more similar ($\Delta p{F}_{YAN}$ = 0.071 ± 0.088 and $\Delta t{F}_{YAN}$ = 0.060 ± 0.032, and $\Delta p{F}_{YAN}$ = 0.071 ± 0.083 and $\Delta t{F}_{YAN}$ = 0.058 ± 0.031, respectively). In spite of the similar computation methods applied, mean $\Delta t{F}_{i}$ values computed for ${F}_{ROH}$, ${F}_{HRR}$, and ${F}_{LH}$ were lower than the genealogical increase in inbreeding (0.066 ± 0.048), which, in turn, was closer to the BP-adjusted and jackknifed mean values of $\Delta t{F}_{YAN}$. These trends were the same for the $t$-subset. Figure 4 shows the variation in the genealogical increase in inbreeding and both the individual increase in homozygosity and the increase in pairwise homozygosity by pedigree depth. As expected, for $t$ ≥ 2, the $\Delta t{F}_{i}$ values tended to fit the genealogical reference ($\Delta {F}_{i}$) better than the $\Delta p{F}_{ijk}$ values. Furthermore, while $\Delta t{F}_{i}$ values tended to be lower than the mean genealogical increase in inbreeding, particularly for $\Delta t{F}_{LH}$, the $\Delta p{F}_{ijk}$ values could take higher or lower values than the genealogical reference at different levels of $t$.

Consistent with the lower mean homozygosity values computed via jackknifing over autosomes, mean jackknifed $\Delta t{F}_{i}$ values were slightly lower than those computed using BP-adjusted values (Table 1). However, the increase in pairwise homozygosity values showed the opposite pattern, with mean jackknifed $\Delta p{F}_{ijk}$ values higher than the corresponding BP-adjusted values, except for ${F}_{YAN}$. In practical terms, the mean values for both $\Delta t{F}_{YAN}$ and $\Delta p{F}_{YAN}$ were the same.

### Effective population size

Table 2 gives the effective population size computed from genealogical and genomic information for the five yearly and genealogical cohorts defined. Regarding genealogical information, *N*_*e*_ computed from individual increases in inbreeding was slightly higher than that computed from increases in pairwise coancestry, except for C2007 (which was approximately twofold higher) and CG3, for which ${N}_{e}{C}_{ij}$ (7.8) was higher than ${N}_{e}{F}_{i}$ (7.0).

**﻿Table 2:**
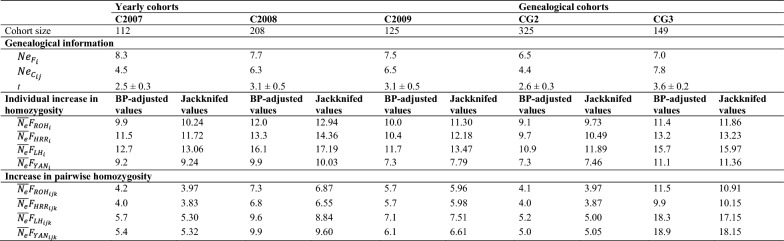
Estimates of effective population size computed using genealogical information (individual increase in inbreeding, ${Ne}_{{F}_{i}}$_*i*_, and coancestry, ${Ne}_{{C}_{ij}}$) and genomic information (individual increase in homozygosity and increase in pairwise homozygosity)

Increases in genomic homozygosity were computed for four different genomic parameters: ${F}_{ROH}$, ${F}_{HRR}$, ${F}_{LH}$ and ${F}_{YAN}$. Genomic-based estimates of *N*_*e*_ were computed from raw homozygosity values as well as from unbiased estimates obtained via jackknifing over autosomes. Estimates were computed for the five yearly and genealogical cohorts defined. Mean equivalent discrete generations (*t*; standard deviation in brackets) are also given

The *N*_*e*_ values estimated using jackknifed individual increases in homozygosity were always higher than those obtained after adjustment for the BP only. However, the opposite pattern was observed for the *N*_*e*_ estimates based on the increase in pairwise homozygosity. Estimates of *N*_*e*_ computed using $\Delta p{F}_{ijk}$ tended to be roughly half of those computed using $\Delta t{F}_{i}$. In any case, independently of the method for the calculation of increases in homozygosity, estimates of $\overline{{N }_{e}}{F}_{{YAN}_{ijk}}$ tended to be nearer to their $\overline{{N }_{e}}{F}_{{YAN}_{i}}$ counterparts.

*N*_*e*_ estimates obtained using ${F}_{YAN}$ had stable root-mean-squared errors, independent of the approach used for computation of the increase in homozygosity (Table 3). However, when an increase in pairwise homozygosity was considered, $\overline{{N }_{e}}{F}_{{ROH}_{ijk}}$ and particularly $\overline{{N }_{e}}{F}_{{HRR}_{ijk}}$ resulted in lower RMSE values.

**Table 3:** Root-mean-squared error (RMSE) for the genomic-based *N*_*e*_ estimated for the five yearly and genealogical cohorts defined using the corresponding ${Ne}_{{F}_{i}}$ and ${Ne}_{{C}_{ij}}$ values as references

	${Ne}_{{F}_{i}}$		${Ne}_{{C}_{ij}}$	
	BP-adjusted values	Jackknifed values	BP-adjusted values	Jackknifed values
Individual increase in homozygosity				
$\overline{{N }_{e}}{F}_{{ROH}_{i}}$	3.2	3.3	3.0	3.1
$\overline{{N }_{e}}{F}_{{HRR}_{i}}$	3.4	3.5	3.2	3.3
$\overline{{N }_{e}}{F}_{{LH}_{i}}$	3.7	3.8	3.4	3.5
$\overline{{N }_{e}}{F}_{{YAN}_{i}}$	3.0	3.0	2.8	2.8
Increase in pairwise homozygosity				
$\overline{{N }_{e}}{F}_{{ROH}_{ijk}}$	2.6	2.5	2.5	2.5
$\overline{{N }_{e}}{F}_{{HRR}_{ijk}}$	2.5	2.5	2.4	2.4
$\overline{{N }_{e}}{F}_{{LH}_{ijk}}$	3.0	3.0	2.9	2.9
$\overline{{N }_{e}}{F}_{{YAN}_{ijk}}$	3.0	3.0	2.9	2.9

## Discussion

Three groups of inbreeding coefficients were assessed in this study: (a) genealogical ${F}_{i}$, as a standard indicator of the genetic variability in a pedigree; (b) ${F}_{ROH}$ and ${F}_{HRR}$, estimating genomic identity from homozygous and heterozygous genomic segments, respectively; and (c) ${F}_{LH}$ and ${F}_{YAN}$, estimating homozygosity while accounting for the allele frequencies in the BP. The pedigree-based inbreeding coefficients reflect IBD probabilities at an infinite number of unlinked loci over descendants that share a pedigree. However, this assumption does not fit with genomic inbreeding estimates. It is unlikely that individuals of a founder population, for instance, in populations under conservation programmes [58], do not show different degrees of genomic relatedness and, possibly, some degree of structuring. This is why the definition of a BP for adjustment of genomic inbreeding estimates is challenging [13].

### Usefulness of estimates of homozygosity

Comparison among homozygosity parameters is not straightforward, not only because of differences in computation but also because they are dependent on the variability in the BP [13, 15]. The probabilistic process of Mendelian segregation results in large variance across loci, among individuals, and at the population level. Consequently, homozygosity parameters have higher variation than expected from genealogies, even in full sibs [19, 48]. The four homozygosity parameters used here were adjusted for the mean homozygosity values in the BP, making the different estimates more comparable (Fig. 2).

The first group of homozygosity parameters tested, *F*_*ROH*_ and *F*_*HRR*,_ are not computed using allele frequencies [59]. Their main advantage is that, before BP adjustment, their definition always yields positive values (ranging from 0 to 1), leading to a straightforward interpretation. However, technical issues, such as marker density and the computational methods used (and the parameters assumed) for identification of either ROH or HRR segments, affect the estimates due to a possible less-efficient identification of shorter segments. This may be of importance for both ${F}_{ROH}$ and ${F}_{HRR}$. Since we used SNP array data with sufficient density to capture long homozygous or heterozygous segments, we applied the consecutive runs approach to identify both ROH and HRR stretches. This approach has been reported to have better performance for the identification of short (homozygous or heterozygous) genomic stretches than the well-known sliding window approach [17, 60].

Different studies have suggested that ${F}_{ROH}$ can be an accurate estimator of IBD [13, 16, 61–63]. However, in small pedigrees, ${F}_{ROH}$ may depend to a large extent on long ROH segments [6]. ROH patterns in a population are frequently conditioned by either recent demographic events or selection in the populations under study, leading to the occurrence of long ROH segments [63, 64]. Homozygosity estimates are greater when using long ROH segments than when using shorter ROH segments [13]. Therefore, fitting well with the Gochu Asturcelta scenario analysed, ${F}_{ROH}$ better characterizes recent inbreeding than old inbreeding.

This is the first study that uses ${F}_{HRR}$ to estimate genomic inbreeding. Unlike ROH, the nature of HRR is not well understood. Although recombination and other processes can shape the frequency and distribution of ROH over the genome, ROH are significantly more common in regions with high linkage disequilibrium and low recombination, tending to be identically inherited from the parents and therefore provide a good IBD estimator (see Gibson et al. [65] and many others later). Although the occurrence of HRR has been reported to be statistically non-random and to cluster in specific chromosomal regions [48], it seems to be independent from linkage disequilibrium in the regions [66]. Furthermore, HRR have been recently shown to be independent of the expected heterozygosity in the population [67] and informative about population structure and demographic history [49].

Altogether, the published evidence described above suggests that the evolutionary forces that shape ROH and HRR segments can be different. Furthermore, our results (Fig. 1) confirm that the chromosomal region with ROH and HRR segments do not overlap. Although the usefulness of HRR for monitoring genomic diversity in livestock needs further confirmation, we consider that the information provided by HRR through the different evolutionary forces acting on different chromosomal regions cannot be disregarded and is likely to be, at least, complementary to that provided by ROH. In any case, the increasing availability of sequence data will further clarify the evolutionary and demographic drivers of HRR and the role of these regions in livestock adaptation.

The literature suggests that HRR are short or very short and that they could be subject to balancing or countervailing selection [17, 44, 48, 49]. Our results confirm that HRR segments are short (always shorter than 1 Mb, and 89% of them shorter than 100 kb). Therefore, theoretically, the ability of ROH and HRR to characterize overall homozygosity (gain of homozygous stretches or loss of heterozygous stretches) in an individual may differ. However, our results suggest that after BP adjustment, both ${F}_{ROH}$ and ${F}_{HRR}$ values are highly consistent (Table 1; Fig. 2).

In contrast with ${F}_{ROH}$ and ${F}_{HRR}$, which are putatively useful to characterize the overall homozygosity in an individual or population, the second group of homozygosity parameters, ${F}_{LH}$ and ${F}_{YAN}$, have been reported as useful estimators of homozygosity due to IBS [9, 16, 45, 53, 59]. The results of ${F}_{LH}$ and ${F}_{YAN}$ depend on the allele frequencies of a defined reference population [45]. Furthermore, ${F}_{YAN}$ is heavily weighted by the frequency of rare alleles [13, 15, 53, 59]. Values obtained for either ${F}_{LH}$ or ${F}_{YAN}$ can range from -1 to 1, and in our data, individual estimates of ${F}_{LH}$ and ${F}_{YAN}$ took negative values with some frequency. Caballero et al. [13] showed that both ${F}_{LH}$ and ${F}_{YAN}$ can take lower values than expected due to the excess of heterozygotes expected for markers in finite populations. Therefore, the behaviour of ${F}_{LH}$ and ${F}_{YAN}$ cannot be straightforwardly compared to that of ${F}_{ROH}$ and ${F}_{HRR}$ [15, 53] unless BP adjustment is applied. Although ${F}_{ROH}$,${F}_{HRR}$, and ${F}_{LH}$ weight each genotyped allele equally, Yang et al.’s [54] method tends to yield higher homozygosity estimates in individuals that are homozygous for rare alleles [13, 15]. Other methods, particularly ${F}_{ROH}$, were less efficient in capturing homozygosity of rare alleles [52].

### Adjustment of homozygosity estimates

The reasons summarized above make different homozygosity parameters difficult to compare. To overcome this issue, we adjusted the four homozygosity measures according to the mean values in the BP, allowing more comparable behaviours among them. However, estimates of homozygosity are strongly affected by Mendelian sampling and the genomic relatedness between individuals in the BP [8]. The adjustment for variability in the BP does not allow complete correction of the estimates (Fig. 2). In this scenario, outliers can still occur, therefore suggesting that additional strategies (here, ‘jackknifing over autosomes’) should be applied to improve robustness of the estimates.

After correction for the mean homozygosity in the BP, the main differences in behaviour were found for ${F}_{YAN}$, confirming that this parameter is highly sensitive to this adjustment. There is no consensus on how to best define the BP. Some authors reported that using the actual allele frequencies in the BP can give inconsistent ${F}_{YAN}$ values across generations with unrealistic patterns of increases or decreases in variability [15]. Other authors reported that fixing the allele frequencies in the base population to 0.5 [8, 68] allows stronger correlations of genealogical inbreeding estimates with genomic estimates computed from the genomic relationship matrix to be obtained. However, defining the current population as the BP yields variable correlations, even negative, with pedigree-based estimates [13].

Furthermore, lack of information about the real founders in a population can bias estimates of inbreeding parameters. This is particularly important when the aim is to compare estimates of homozygosity and inbreeding because the number of loci genotyped is always finite and the base (or founder) population has a different proportion of homozygous loci assumed to be IBS, whereas when using genealogies, founders are assumed to be unrelated and *F* is always dependent on the pedigree depth [69, 70]. Although small in size, our BP included two out of four of the founders of the population of Gochu Asturcelta and four direct descendants of the non-genotyped founders [32, 33]. Estimates of inbreeding that account for allele frequencies (${F}_{LH}$ and ${F}_{YAN}$) are expected to capture relationships due to the existence of common ancestors in a population, tracing the genetic variability of the present population back to the BP [16]. However, the literature suggests that founder allele frequencies in small populations have little meaning: in the absence of mutation, all current copies of an allele may be IBD, and its current frequency simply represents the reproductive success of the founder and its descendants [45] and could be related to the census size of breeders [13]. In our data, homozygosity tended to increase significantly with pedigree depth ($t$), and BP adjustment resulted in acceptably consistent behaviour among homozygosity parameters (Fig. 3; Table 1). However, this approach may not be sufficient and jackknifing over autosomes suggested that additional adjustments would be necessary to obtain robust estimates; thus, BP-adjusted estimates of homozygosity may be biased upwards.

In general, our results suggest that BP adjustment does not remove differences in performance among homozygosity parameters, therefore suggesting that the genetic variability of a population should be assessed with caution. Of course, allele frequencies may be strongly affected by both editing of genotype data and the presence of allelic dropouts and null alleles in arrays [37], which can bias homozygosity estimates. Removal of rare alleles can cause an apparent increase in inbreeding due to IBS rather than IBD [9, 62]. To avoid this undesirable effect, we edited the SNP array data, filtering only loci that have Mendelian errors only, which have been previously shown to be mainly caused by technical issues and cause spurious diversity [37].

### Demographic causes of variation in genomic estimates of homozygosity

In our population, homozygosity did not show clear trends of variation in relation to pedigree depth (Fig. 3). In fact, the accumulation of homozygosity with pedigree depth in inbred populations may be lower than expected when minimum coancestry matings are implemented, as in our case [32]. The Gochu Asturcelta conservation programme applied a strict mating policy to avoid matings between close relatives and to prolong the reproductive career of the founders and their direct descendants as much as possible to keep founder contributions balanced across generations and to preserve their genetic background in the population [32]. Theoretical and empirical evidence suggests that mating policies involving minimum coancestry in undivided populations balance allele frequencies at neutral genetic markers across generations [18, 23], therefore minimizing Mendelian segregation variance [22].

Although genomic estimates of inbreeding reflect the percentage of the genome that is homozygous, pedigree-based estimates are only expectations [20]. This suggests that the ability of homozygosity parameters to characterize the genetic variability of a population should be tested in each particular case, making it difficult to provide general guidelines. The results obtained in a particular population may be shaped by its particular breeding scenario. The literature suggests that between-individual variation in inbreeding estimators is strongly increased by presence of population structure [45] as well as by relatedness within the population, the mating policy applied, and the presence of large families increase [13, 58].

### Increases in homozygosity and effective population size

Here, we evaluated two approaches to compute the increase in homozygosity. All estimates had large standard deviations (Table 1) as a consequence of the relatively small sample size and the strong effect of Mendelian sampling on the estimates of genomic inbreeding. The individual increase in homozygosity is conceptually similar to the individual increase in inbreeding ($\Delta {F}_{i}$) proposed by Gutiérrez et al. [27, 28], which has been used as a standard for comparisons. Although $\Delta {F}_{i}$ allows a flexible definition of the reference population and is independent of mating policies [29, 41], it is based on the assumption that losses in diversity depend on pedigree depth only [71]. However, this assumption cannot be straightforwardly applied to individual increases in homozygosity: although founder individuals and their direct descendants in a pedigree are not inbred, all individuals in a pedigree have different degrees of homozygosity that can partially be explained by IBS. Our data suggest that the accumulation of the pedigree tends to increase homozygosity (Fig. 3). However, matings between relatives may be more important. In this regard, the increase in pairwise homozygosity could be intuitively considered a more accurate estimate of the increase in homozygosity due to IBD. In any case, increases in homozygosity are not constant across the genome [72]. IBD estimates in a limited genomic area or at the genome-wide level exhibit more variance than those computed for chromosomes [5]. To avoid this, jackknifing over autosomes was applied to the BP-adjusted genomic inbreeding estimates.

Although the differences were small, the results obtained after jackknifing suggest that BP-adjusted estimates of individual increases in homozygosity can be overestimates, while BP-adjusted values for increases in pairwise homozygosity can be underestimates (Table 1; Fig. 4). Values obtained for $\Delta t{F}_{i}$ displayed consistent variation with $\Delta {F}_{i}$, which can be explained by their similar computation methods. The two approaches tested for increases in homozygosity showed consistent behaviour for ${F}_{ROH}$*,* ${F}_{HRR}$, and ${F}_{LH}$. However, the increases in homozygosity computed using ${F}_{YAN}$ departed from this general behaviour. ${F}_{YAN}$ is expected to have a smaller sampling variance than other estimates of homozygosity [54]. Our results confirmed this, as $\Delta t{F}_{YAN}$ had lower variation (54.1%) than $\Delta t{F}_{HRR}$ (105.9%).

The usefulness of the two approaches tested for increases in homozygosity to compute *N*_*e*_ has been compared with that of the genealogical “realized” *N*_*e*_ computed from $\Delta {F}_{i}$ but also with that derived from the increase in pairwise coancestry proposed by Cervantes et al. [73]. The last approach is expected to give more stable estimates of *N*_*e*_ in cases of shallow pedigrees due to the larger amount of information used in the computations.

Estimates of *N*_*e*_ obtained from genealogical and genomic information are expected to have large differences [16, 22, 68, 74, 75]. Indeed, criteria based on pedigree information refer to an infinite number of loci [71], while criteria based on observed genomic polymorphism mirror phenomena related to temporal changes in allele frequency at a limited number of loci [22, 76]. Furthermore, genealogical estimates of* N*_*e*_, frequently used as a reference in the literature, may not reflect the actual *N*_*e*_. Even with suitable genealogical data, genealogical *N*_*e*_ does not consider natural selection against homozygotes. Therefore, the genetic variability at the genomic level could be higher than expected. Although different genomic methods have been proposed to obtain reliable *N*_*e*_ estimates using half- and full-sib allele frequencies [77] and heterozygote excess [78], computation of reliable estimates of *N*_*e*_ is still a challenge. Mendelian sampling causes large differences in allele frequencies among close relatives (e.g., full-sibs) and at the population level. This may be particularly important in small populations with shallow pedigrees and frequent matings between relatives.

Genomic approaches are expected to give higher estimates of *N*_*e*_ than approaches based on genealogical data [14, 33], probably varying with the actual number of breeders in the sample [13]. This expectation fits well with the estimates of *N*_*e*_ obtained using individual increases in homozygosity. However, this is not so clear for increases in pairwise homozygosity. The assumption that an increase in homozygosity only depends on pedigree depth causes $\Delta t{F}_{i}$ values to underestimate the IBD scenario of the subpopulations analysed when compared with $\Delta p{F}_{i}$. Our results suggest that pedigree depth itself may influence the accumulation of homozygosity (see Additional file 1: Table S3). However, this effect may be weaker than that of matings between relatives. In any case, jackknifing over autosomes can help avoid under- and overestimation of *N*_*e*_. For individual increases in homozygosity, estimates of $\overline{{N }_{e}}{F}_{{YAN}_{i}}$ tended to be closer to both ${N}_{e}{F}_{i}$ and ${N}_{e}{C}_{ij}$ estimates. However, this was not the case for the increase in pairwise homozygosity, which was more similar to $\overline{{N }_{e}}{F}_{{ROH}_{ijk}}$ and, particularly, to $\overline{{N }_{e}}{F}_{{HRR}_{ijk}}$. The lower variance of the ${F}_{YAN}$ values may lead to better adjustment of the influence of pedigree depth on increases in homozygosity.

Estimates of *N*_*e*_ based on $\Delta p{F}_{ROH}$ and, particularly, on $\Delta p{F}_{HRR}$ had lower RMSE values in the dataset. If $\Delta p{F}_{ijk}$ values are useful for characterizing increases in homozygosity due to IBD, our results suggest that increases in homozygosity would be caused by losses of small HRR genomic segments, which could be more sensitive to mating between relatives than to the increase in the length of long stretches of ROH.

In any case, as expected, estimates of genomic *N*_*e*_ were affected by cohort size: the larger the subpopulation assessed is, the higher the estimates of genomic *N*_*e*_. In this regard, the values for the cohorts C2008 and CG3 had the highest estimates for a given method. The yearly cohort C2008 included several non-inbred individuals (*F* = 0), which could lead to higher *N*_*e*_ estimates. However, this did not happen in CG3, therefore confirming some dependency on genomic *N*_*e*_ and sampling size. In the Gochu Asturcelta population, cohort sizes have been reported to be crucial for obtaining reliable estimates of *N*_*e*_ [33]. Our results, obtained using completely different information and methods, confirm this point.

## Conclusions

Here, we analysed the ability of different genomic estimates of homozygosity to characterize the genetic background of a livestock population under mating policies aiming at conserving genetic variability. Our study showed that the mating policy applied to the Gochu Asturcelta pig population was successful in maintaining balanced allele frequencies. However, in such scenarios, parameters that characterize homozygosity are limited in their ability to depict trends in losses of variability. In spite of the presence of closely-related individuals from different families that vary in size, the four homozygosity parameters used (${F}_{ROH}$, ${F}_{HRR}$, ${F}_{LH}$, and ${F}_{YAN}$) characterized the evolution of the pedigree to some extent. However, different adjustments were necessary to improve robustness of the estimates: although the adjustment of the homozygosity values by the mean of the BP appears indispensable, additional adjustment via jackknifing over autosomes helps to account for within-individual genome variation. Unlike genealogical approaches, the assumption that increases in homozygosity depend on pedigree depth only may not apply to small, shallow pedigrees, and an increase in pairwise homozygosity may better characterize autozygosity. Although increases in homozygosity computed using ${F}_{YAN}$ seem to have good general behaviour, increases in pairwise homozygosity computed using ${F}_{ROH}$ and ${F}_{HRR}$ may be particularly useful if efficient identification of short ROH and HRR segments can be ensured.

### Supplementary Information

**Additional file 1**: **Table S1.** Pedigree used and raw individual estimates of homozygosity. Description of the pedigree of the Gochu Asturcelta pigs used and raw estimates of homozygosity parameters computed at the individual level. **Table S2:** BP-adjusted homozygosity estimates. Description of the BP-adjusted homozygosity values and the corresponding increase in homozygosity parameters computed at the individual level. **Table S3:** Regression of homozygosity against equivalent discrete generations. Regression of four homozygosity estimates and their corresponding individual increase in homozygosity (Δ*t*) and paired increase in homozygosity (Δ*p*) against equivalent discrete generations (*t*). **Table S4:** Jackknifed homozygosity values and their corresponding individual increases in homozygosity (Δ*t*) and paired increases in homozygosity (Δ*p*) values. Description of the jackknifed homozygosity values and the corresponding increase in homozygosity parameters computed at the individual level.

## Data Availability

All data necessary to replicate the results presented are provided as Additional Tables (.xlsx). SNP array data are currently under analysis within the “AutoGenome” project framework. However, the dataset used is available from the corresponding author upon reasonable request.
